# Resonance-stabilized partial proton transfer in hydrogen bonds of incommensurate phenazine–chloranilic acid

**DOI:** 10.1107/S2052520615004084

**Published:** 2015-03-31

**Authors:** Leila Noohinejad, Swastik Mondal, Sk Imran Ali, Somnath Dey, Sander van Smaalen, Andreas Schönleber

**Affiliations:** a Laboratory of Crystallography, University of Bayreuth, 95440 Bayreuth, Germany; b Max-Planck-Institut für Kohlenforschung, Kaiser-Wilhelm-Platz 1, 45470 Mülheim an der Ruhr, Germany

**Keywords:** protron transfer, ferroelectric materials, incommensurate structure

## Abstract

The co-crystal of phenazine (Phz) and chloranilic acid (H_2_ca) becomes ferroelectric upon cooling through the loss of inversion symmetry. Further cooling results in the development of an incommensurate ferroelectric phase, followed by a lock-in transition towards a twofold superstructure. Here we present the incommensurately modulated crystal structure of Phz-H_2_ca at *T* = 139 K with a symmetry given by the superspace group *P*2_1_(½ σ_2_ ½)0 and σ_2_ = 0.5139. The modulation mainly affects the positions of the protons within half of the intermolecular hydrogen bonds that are responsible for the spontaneous polarization in all three low-temperature phases. Evidence for proton transfer in part of the hydrogen bonds is obtained from the correlated dependence on the phase of the modulation of the lengths of bonds involved in resonance stabilization of the acidic anion, and much smaller variations of bond lengths of atoms not involved in the resonance mechanism. Incommensurability is explained as competition between proton transfer favored for single hydrogen bonds on the basis of p*K*_a_ values and avoiding unfavorable Coulomb repulsion within the lattice of the resulting ionic molecules.

## Abstract 2

Correlated variations of chemical bonds demonstrate stabilization by the resonance of the chloranilic acid anion. Proton transfer in some of the intermolecular hydrogen bonds is responsible for the ferroelectic properties.

## 1. Introduction   

Organic compounds are of interest as ferroelectric materials, because they have a low density and they are potentially cheap to produce (Horiuchi & Tokura, 2008 ▶). Furthermore, organic compounds offer more possibilities than inorganic compounds for designing properties. Organic materials based on hydrogen-bonded supramolecular chains with a polar space group form one class of ferroelectric materials. The co-crystal of phenazine (Phz) and 2,5-dichloro-3,6-dihydroxy-*p*-benzoquinone (chloranilic acid, H_2_ca) is one of several recently discovered hydrogen-bonded organic ferroelectrics (Horiuchi, Ishii *et al.*, 2005 ▶; Horiuchi *et al.*, 2009 ▶; Kumai *et al.*, 2006 ▶, 2012 ▶).

Phz-H_2_ca contains chains of alternating Phz and H_2_ca molecules connected through O—H⋯N intermolecular hydrogen bonds. At room temperature, all hydrogen bonds are equivalent by the symmetry of the centrosymmetric space group 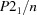 (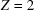), and the crystal of Phz-H_2_ca is paraelectric (PE phase; Horiuchi, Ishii *et al.*, 2005 ▶; Kumai *et al.*, 2007 ▶). Below 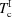 = 253 K the symmetry is reduced to 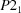 (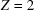), allowing for two inequivalent hydrogen bonds. One of the two bonds exhibits partial proton transfer, which is responsible for the spontaneous polarization (FE-I phase; Horiuchi, Ishii *et al.*, 2005 ▶; Kumai *et al.*, 2007 ▶; Gotoh *et al.*, 2007 ▶). Phz-H_2_ca has an incommensurately modulated structure between 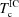 = 147 K and 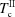 = 137 K (FE-IC phase; Saito *et al.*, 2006 ▶; Horiuchi *et al.*, 2009 ▶). Below 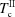 another ferroelectric phase is stable that can be characterized as a twofold superstructure of the room-temperature structure (FE-II phase; Noohinejad *et al.*, 2014 ▶).

Here we report the crystal structure of the incommensurate phase, employing the superspace formalism applied to single-crystal X-ray diffraction data. The modulation is found to mainly affect the positions of the H atoms within the O—H⋯N intermolecular hydrogen bonds. Evidence for proton transfer in part of these bonds is provided by the correlated variations of bond lengths reflecting resonance stabilization of the anion. A detailed comparison of the various phases reveals that the incommensurate phase has a crystal structure intermediate between the crystal structures of the FE-I and FE-II phases. A mechanism is proposed for the sequence of phase transitions.

## 2. Experimental   

### 2.1. X-ray diffraction   

Single crystals of Phz-H_2_ca were obtained by cosublimation of phenazine and chloranilic acid (Horiuchi. Ishii *et al.*, 2005 ▶; Noohinejad *et al.*, 2014 ▶). A diffraction experiment at *T* = 139 K was performed on the same crystal as was employed in our previous study on the commensurate FE-II phase (Noohinejad *et al.*, 2014 ▶). X-ray diffraction data have been measured at beamline F1 of Hasylab at DESY in Hamburg, Germany, employing a MAR165 CCD detector mounted on a kappa diffractometer. The temperature of the crystal was regulated by a nitrogen gas-flow cryostat. X-ray diffraction data were collected by ϕ scans and ω scans for various settings of the orientation of the crystal. To better evaluate strong and weak reflections, two measurements were performed with the same measurement strategies but with different exposure times of 20 and 160 s, respectively. Data processing of the measured images has been carried out with the software *EVAL*15 (Schreurs *et al.*, 2010 ▶) to index and extract the integrated intensities of Bragg reflections, and with *SADABS* (Sheldrick, 2008 ▶) for absorption correction. The latter employed groups of equivalent reflections defined according to the point group 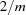, which appeared as the symmetry of the diffraction. Experimental details are given in Table 1 ▶.

Indexing of the diffraction images with *EVAL*15 resulted in an indexing with four integers on the basis of a monoclinic unit cell closely related to the unit cell of the FE-I phase at 160 K (Horiuchi, Ishii *et al.*, 2005 ▶) together with the incommensurate modulation wavevector 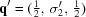, where 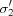 = 0.4861. However, the integration routine of *EVAL*15 did not accept a modulation wavevector with rational components. Therefore, the integration has been performed within the supercentered setting with 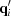 = 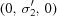 and centering translation 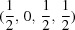 with respect to the transformed basic structure unit cell 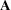 = 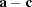, 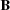 = 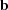, and 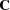 = 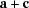 (Stokes *et al.*, 2011 ▶). The same setting has been employed in *SADABS*.

### 2.2. Choice of the superspace group   

The low-temperature superstructure of the FE-II phase at 100 K has been described as a commensurately modulated structure with a basic structure similar to the structure at higher temperatures and the commensurate modulation wavevector 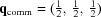. The superspace group 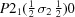, with 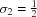 has been found to describe the symmetry of this phase (Noohinejad *et al.*, 2014 ▶).

Presently, the indexing with modulation wavevector 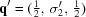 and 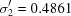 (see §2.1) leads to the superspace group 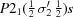. The non-centrosymmetric superspace group is established by the lack of inversion symmetry of both the FE-I and FE-II phases (see §1) as well as by measurements of the electrical polarization, indicating a ferroelectric state below 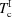 (Horiuchi, Kumai & Tokura *et al.*, 2005 ▶). The two superspace groups appear to be alternate settings of superspace group No. 4.1.6.3 with standard setting 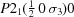 (Stokes *et al.*, 2011 ▶). The two settings can be transformed into each other by a shift of the origin. However, this would result in different coordinates of the atoms in the basic structures of the low-temperature and incommensurate phases, which is not desired. The setting with zero intrinsic translation along the fourth coordinate can also be obtained by the choice of a different modulation wavevector for the incommensurate modulation, according to
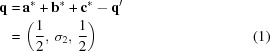
with
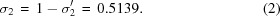



Diffraction data were re-indexed according to this transformation [equations (1) and (2)], and the superspace group 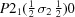 with 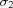 = 0.5139 has been used for all refinements.

### 2.3. Structure refinements   

Initial values for the parameters of the basic structure have been taken from the basic structure at 100 K (Noohinejad *et al.*, 2014 ▶). Anisotropic atomic displacement parameters (ADPs) have been used for all non-H atoms. H atoms were placed at calculated positions with a bond length *d*(C—H) of 0.96 Å, and they were refined using a riding model with isotropic ADPs equal to 1.2 times the equivalent isotropic ADPs of the bonded C atoms. H atoms of the hydroxyl groups were located in the difference Fourier map. They were then shifted to positions fulfilling the restraints *d*(O—H) = 0.85 (2) Å and ∠(C—O—H) = 109.5 (2)° (Müller *et al.*, 2006 ▶; Engh & Huber, 1991 ▶), while their isotropic ADPs were restricted to 1.5 times the equivalent isotropic ADPs of the adjacent O atoms. Employing *JANA*2006 (Petricek *et al.*, 2014 ▶), the positions of all atoms were refined with these restraints in effect. In the last step the restraints were released, resulting in a good fit to the main reflections with *R*_obs_ = 0.0412.

Three approaches have been chosen for determination of the atomic modulation functions for the incommensurate phase. In one approach, the modulation functions of the model at 100 K (Noohinejad *et al.*, 2014 ▶) were used as a starting model. The same superspace group was employed, but with 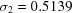 instead of the commensurate value of 0.5. The refinement converged smoothly to a good fit to the combined set of main and satellite reflections, resulting in model A (Table 1 ▶). Model A involves one harmonic wave for the displacive modulation of all atoms as well as one harmonic wave for the modulation of ADPs of all non-H atoms. The origin was fixed on the Cl2 atom. Inversion twins are expected to be present, because the IC phase has been reached by phase transitions, starting with the centrosymmetric PE phase at room temperature. Twinning did have a marginal effect on the refinement, while a significant deviation from equal volume fractions of the twin domains was not found. Therefore, equal volume fractions were employed for the final refinements. A model with the alternative symmetry 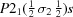 did not lead to a good fit to the data.

Starting with the same basic structure, model B was developed by assigning arbitrary but small values to the modulation parameters of the heaviest atom (chlorine). Refinements alternated with the subsequent introduction of modulation parameters for the O, N, C and H atoms, finally resulting in a fit to the diffraction data of equal quality as that of model A (Table 2 ▶).

**Figure 1:**
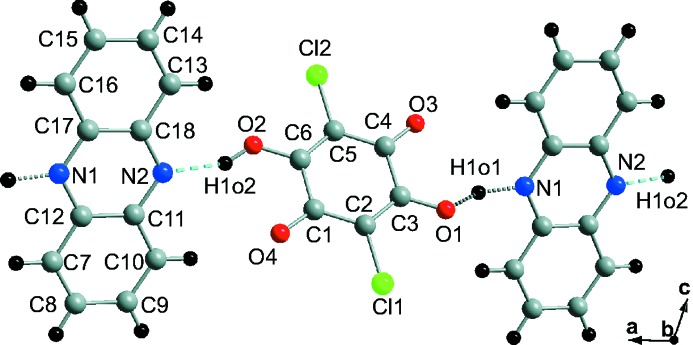
Phenazine C_12_H_8_N_2_ and chloranilic acid C_6_Cl_2_H_2_O_4_ with the atom labels as employed in the present work.

In a completely different approach, charge flipping was applied for the direct solution of the incommensurately modulated structure in superspace (Palatinus & Chapuis, 2007 ▶; Palatinus, 2013 ▶). For the solution, the software *SUPERFLIP* suggested the centrosymmetric symmetry 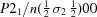. Since we knew that the modulation is non-centrosymmetric, we have chosen the superspace group 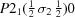. *JANA*2006 was subsequently used to extract the basic structure positions and values of the first-order harmonics of the displacive modulation functions for all non-H atoms. Atoms were then named to match Fig. 1 ▶. For this model the basic structure coordinates were refined against all reflections, resulting in 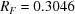, 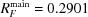 and 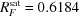. H atoms were added at calculated positions near C atoms as in model A. Refinement of the atomic coordinates within the riding model resulted in 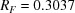, 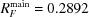 and 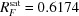. Subsequent refinement of anisotropic ADPs of the non-H atoms resulted in 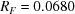, 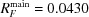 and 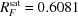. H atoms of the hydroxyl groups were located in the difference-Fourier map and treated as in model A. Refinement of the restrained model gave 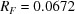, 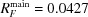 and 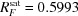. Refinement of the free model gave 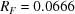, 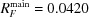 and 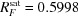. Small values were applied to the displacive modulation functions of the H atoms. Refinement of the modulated structure resulted in 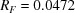, 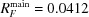 and 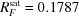. Finally, modulation parameters were introduced for the ADPs of the non-H atoms, resulting in the final fit of model C to the diffraction data as given in Table 2 ▶.

## 3. Discussion   

### 3.1. The structure model   

The final fit to the diffraction data is excellent for the main reflections (Table 1 ▶). The rather high value of 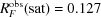 can be completely explained by the weakness of the satellite reflections and the resulting values for 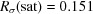, representing the average standard uncertainty over intensity, and 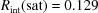 for averaging satellite reflections.

**Figure 2:**
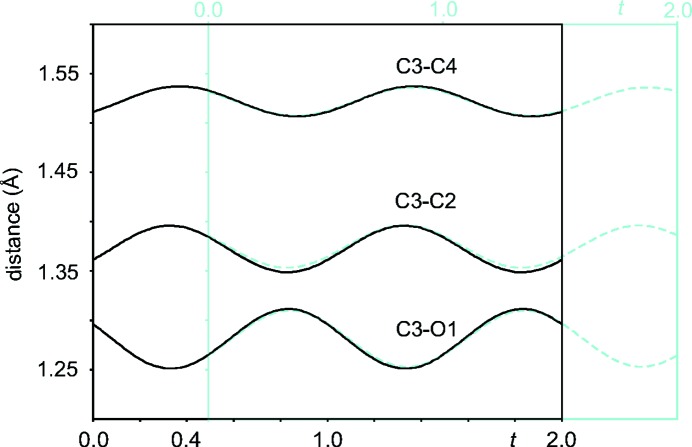
Interatomic distances (Å) as a function of phase *t* of the modulation. The *t* plot for model B (in blue) is superimposed onto the *t* plot for model A (in black), after application of a phase shift of −0.5139 in *t* to model B.

Model A and model B give the same fit to the diffraction data (Table 2 ▶). Although modulation parameters are different, these two models are completely equivalent. They differ from each other by a phase shift (Fig. 2 ▶). Further support for model A comes from difference-Fourier maps obtained after refinements of model A and of a similar model without the acidic H atoms (see the supporting information). Model C has been obtained by solving the modulated structure by charge flipping in superspace. The modulation of model C is different from the modulations in models A and B, but 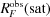 is clearly higher for model C than for the other two models (Table 2 ▶). Therefore, model C provides a less good description of the modulation than models A and B do. Difficulties in obtaining the correct structure model by charge flipping are probably related to the pseudo-symmetry of the structure, with deviations from inversion symmetry being mainly the result of rearrangements of H atoms.

These properties provide strong support that model A (as well as the equivalent model B) is the correct model for the modulated crystal structure of the incommensurate phase. In view of these results, we have restricted the analysis to model A.

### 3.2. Resonance-stabilized proton transfer   

The modulated structure of the incommensurate phase of Phz-H_2_ca has been successfully determined at a temperature of 139 K. The magnitudes of the modulation amplitudes of the atoms reveal that the major effect of the modulation is a displacive modulation of the H atom of one of the two hydrogen bonds in which each molecule is involved in. This feature is in complete agreement with the crystal structures of the FE-I and FE-II phases, where also one half of the hydrogen bond is involved in the distortions of the structure.

**Figure 3:**
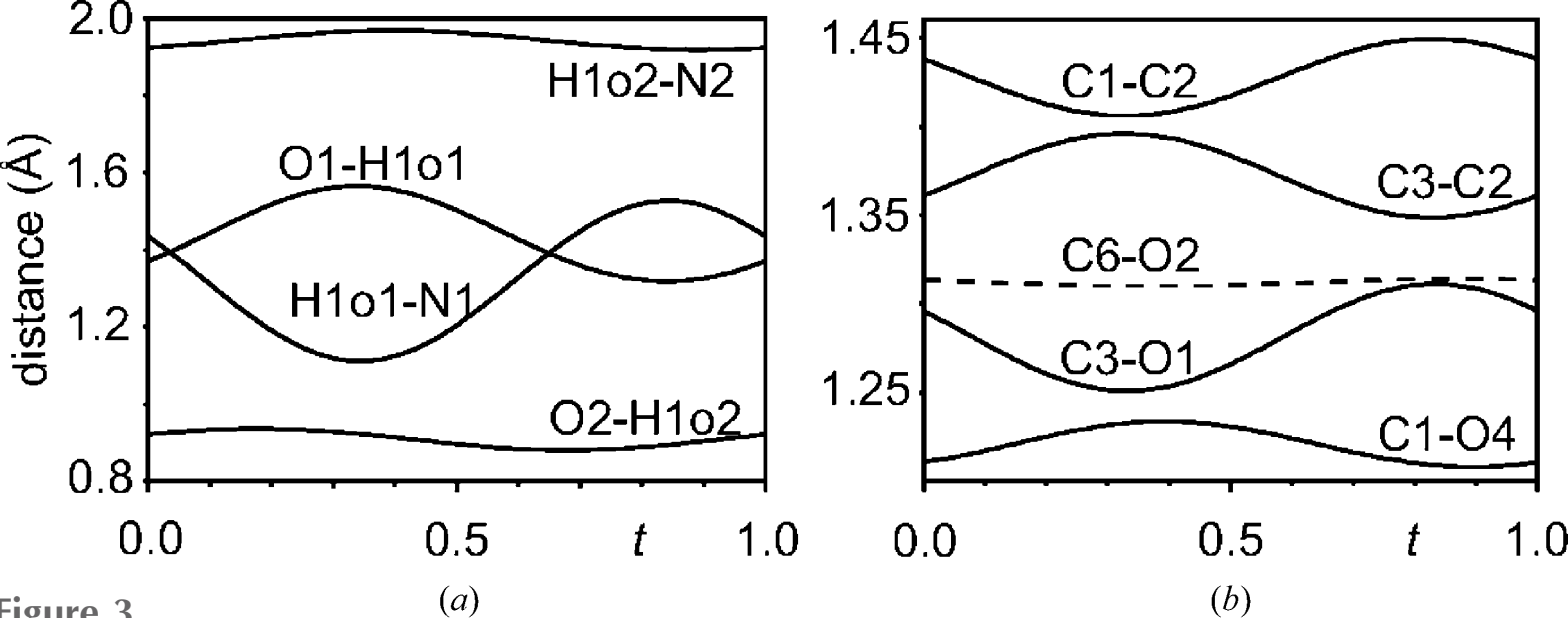
Selected interatomic distances (Å) as a function of phase *t* of the modulation. (*a*) The O—H and N—H distances within the two hydrogen bonds. (*b*) C—C and C—O distances of the resonance system stabilizing the Hca^−^ anion, as well as the C6—O2 distance not involved in resonance. Notice the different length scale on the vertical axes for panels (*a*) and (*b*).

More precisely, the crystal structure of the FE-I phase contains one crystallographically independent molecule H_2_ca with two independent O atoms involved in O—H⋯N hydrogen bonds, denoted by O1 and O2 (Gotoh *et al.*, 2007 ▶). The basic structure of the FE-IC phase is the same as the crystal structure of the FE-I phase, so that the FE-IC phase contains modulated atoms O1 and O2. Finally, the FE-II phase represents a twofold superstructure of the structure of the FE-I phase. Together with a reduction of the point symmetry to triclinic, this results in four crystallographically independent molecules H_2_ca with atoms O1*A* through to O1*D* derived from O1, and atoms O2*A* through to O2*D* derived from O2 (Noohinejad *et al.*, 2014 ▶). In all three phases, the hydrogen bonds O2—H1o2⋯N2 are not involved in superstructure formation. For the FE-IC structure, Table 3 ▶ and Fig. 3 ▶(*a*) show that bond lengths involving the O2, H1o2 and N2 atoms exhibit only a weak dependence on phase *t* of the modulation. For the FE-I and FE-II structures this property has been previously determined by Gotoh *et al.* (2007 ▶) and Noohinejad *et al.* (2014 ▶), and it is summarized in Table 3 ▶. Therefore, the hydrogen bonds O2—H1o2⋯N2 do not play a direct role in the ferroelectricity of this compound.

**Figure 4:**
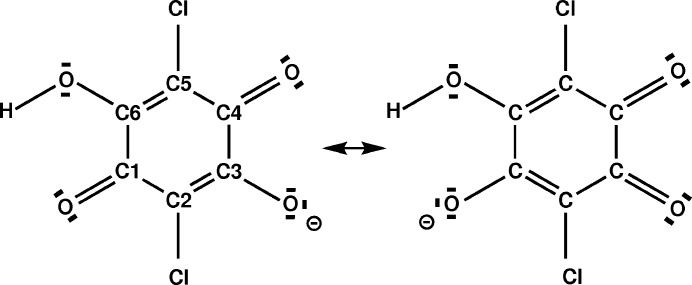
Schematic representation of resonance within the anion Hca^−^ of chloranilic acid.

The largest variation in bond lengths within the FE-IC phase is found for the hydrogen bond O1—H1o1⋯N1, with a variation of Δ*d*(O1—H1o1) = 0.25 Å and Δ*d*(N1—H1o1) = 0.42 Å (Table 3 ▶). All other bonds are much less affected by the modulation, with a maximum variation of 0.06 Å for C3—O1 in H_2_ca and of 0.019 Å for C14—C15 in Phz (see the supporting information). The next largest variations of bond lengths are found for C3—C2, C1—C2 and C1—O4 (Table 4 ▶). These bonds are precisely those involved in resonance stabilization of the Hca^−^ ion, as it is obtained after transfer of the proton within the O1—H1o1⋯N1 hydrogen bond. Further evidence for this interpretation comes from *t*-plots (Fig. 3 ▶), which show that an elongation of the O1—H1o1 bond (interpreted as proton transfer) correlates with an elongation of the C3—C2 and C1—O4 bonds, for which resonance represents the admixture of single-bond character into these formally double bonds (Fig. 4 ▶). Concomitantly, C1—C2 and C3—O1 have become shorter due to the admixture of double-bond character into formally single bonds. A similar variation of bond lengths is found in the crystal structure of the FE-II phase (Table 4 ▶). The results support the model of partial proton transfer (see §3.3).

### 3.3. The ferroelectric phase transitions   

Ferroelectricity in Phz-H_2_ca at low temperatures is the result of intermolecular proton transfer within the O1—H1o1⋯N1 hydrogen bonds (Horiuchi, Kumai & Tokura, 2005 ▶; Kumai *et al.*, 2007 ▶, 2012 ▶; Gotoh *et al.*, 2007 ▶; Noohinejad *et al.*, 2014 ▶). Consideration of the positions of the H atoms within the O1—H1o1⋯N1 hydrogen bonds of the crystal structures of the three phases leads to the following model for the phase transitions.

At room temperature (PE phase) all hydrogen bonds are equivalent by symmetry of the centrosymmetric space group. Consequently, any dipole moment of the O1—H1o1⋯N1 hydrogen bond will be perfectly compensated by a dipole moment of the O2—H1o2⋯N2 hydrogen bond on the same molecule that points in the opposite direction, because O1 and O2 are related by the inversion center. The ferroelectric phase transition towards the FE-I phase is characterized by loss of inversion symmetry. The O2—H1o2 remains short and should be interpreted as a covalent O—H bond that acts as a hydrogen-bond donor towards N2 (Table 3 ▶). The O1—H1o1 bond is clearly elongated compared with a covalent bond, but it is not completely broken. The N1—H1o1 distance is clearly shorter than in the PE phase, but it is not yet the distance of ∼ 1.03 Å of a covalent N—H bond. Therefore, it can be concluded that this structure exhibits partial proton transfer within the O1—H1o1⋯N1 hydrogen bonds.

The low-temperature FE-II has four crystallographically independent O1—H1o1⋯N1 hydrogen bonds. The partial proton transfer of the FE-I phase is replaced in the FE-II phase by complete proton transfer in one half of these hydrogen bonds (B and D), and the absence of proton transfer in the other half (A and C) (Table 3 ▶). The FE-I phase transfers into the FE-II phase *via* the intermediate FE-IC phase. Structurally, the FE-IC phase appears intermediate between the high- and low-temperature ferroelectric phases too. The incommensurate modulations represents a modulation of the O1—H1o1⋯N1 hydrogen bond between one with almost full proton transfer and one which can be characterized as almost no proton transfer (Table 3 ▶ and Fig. 3 ▶ *a*). Despite an incommensurability of the FE-IC phase, it appears that – on average – one quarter of the hydrogen bonds has full proton transfer in both the FE-IC and FE-II phases, while half of the hydrogen bonds in the FE-I phase are affected by partial proton transfer. These similarities might explain the only marginal effects of the ferroelectric incommensurate and lock-in transitions on the macroscopic electric dipole moment (Horiuchi *et al.*, 2009 ▶).

## 4. Conclusions   

The incommensurately modulated structure of the ferroelectric incommensurate (FE-IC) phase of Phz-H_2_ca has been successfully determined. It is shown that this structure is intermediate between the ferroelectric FE-I and FE-II phases. Half of the intermolecular hydrogen bonds exhibit partial proton transfer within the FE-I phase. This becomes an incommensurate variation between strong and very weak proton transfer within the FE-IC phase, while in the FE-II phase the active half of the hydrogen bonds splits into a hydrogen bond with complete proton transfer and one without proton transfer. Strong support for proton transfer in part of the hydrogen bonds has been obtained through the variations of the lengths of precisely those bonds that are involved in resonance stabilization of the Hca^−^ ion (§3.2). Proton transfer in only part of the hydrogen bonds has been explained as the result of Coulomb interactions between the resulting ionic species (Kumai *et al.*, 2012 ▶). Proton transfer is in line with the acidities of the two molecules with p*K*_a1_ = 1.23 for the proton acceptor Phz, and p*K*_a1_ = 0.76 for the proton donor H_2_ca (Albert & Phillips, 1956 ▶; Molcanov & Prodic, 2010 ▶). One could thus suggest that the incommensurability will be the result of competition between the inclination towards proton transfer of single hydrogen bonds and avoiding unfavorable Coulomb repulsion within the crystalline lattice of molecules.

## Supplementary Material


10522E4Avuq

Crystal structure: contains datablock(s) global, I. DOI: 10.1107/S2052520615004084/dk5030sup1.cif

Structure factors: contains datablock(s) block. DOI: 10.1107/S2052520615004084/dk5030Isup2.hkl

Extra tables and figures. DOI: 10.1107/S2052520615004084/dk5030sup3.pdf

CCDC reference: 1051555

## Figures and Tables

**Table 1:** Experimental details

Crystal data	
Chemical formula	C_12_H_8_N_2_C_6_H_2_Cl_2_O_4_
*M*_r_	389.2
Crystal system, superspace group	Monoclinic, *P*2_1_( _2_ )0
Temperature (K)	139
Wavevector	**q** = 0.5a* + 0.5139b* + 0.5c*
*a*, *b*, *c* ()	12.3720(2), 3.7649(5), 16.8315(2)
()	107.789(7)
*V* (^3^)	746.52(14)
*Z*	2
Radiation type	Synchrotron, = 0.56
(mm^1^)	0.24
Crystal size (mm)	0.22 0.13 0.05

Data collection	
Diffractometer	Marresearch, mar165 CCD
Absorption correction	Empirical (using intensity measurements) *SADABS* (Sheldrick, 2008 ▶)
*T*_min_, *T*_max_	0.777, 0.991
No. of measured, independent and observed [*I* > 3(*I*)] reflections	25001, 15433, 8092
Main (obs, all)	5607, 5942
Satellites, first order (obs, all)	2485, 9491
*R*_int_	0.019
(sin /)_max_ (^1^)	0.981

Refinement	
*R*[*F* ^2^ > 2(*F* ^2^)], *wR*(*F* ^2^), *S*	0.045, 0.071, 2.08
No. of reflections	15433
No. of parameters	719
H-atom treatment	H atoms treated by a mixture of independent and constrained refinement
_max_, _min_ (e^3^)	0.17, 0.02
Absolute structure	6649 Friedel pairs used in the refinement
Absolute structure parameter	0.5

**Table 2:** *R* values for the different structure models Included are *R* values for observed (obs; defined by *I* > 3) and all (all) reflections. Partial *R* values are given for the group of main and satellite (sat) reflections. Structure model A is based on the low-temperature commensurate structure; model B has been obtained by refinement with arbitrary but small initial values for the modulation amplitudes; and model C is obtained after refinement of the superflip solution.

Model	A	B	C
GOF^obs^	2.78	2.78	2.79
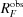 (all)	0.0449	0.0449	0.0451
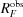 (main)	0.0411	0.0412	0.0411
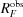 (sat)	0.1268	0.1266	0.1301
GOF^all^	2.08	2.08	2.08
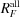 (all)	0.0720	0.0718	0.0720
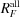 (main)	0.0425	0.0425	0.0425
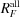 (sat)	0.3661	0.3634	0.3663
No. of parameters	719	719	719

**Table 3:** Geometry of the intermolecular hydrogen bonds O1H1o1N1 and O2H1o2N2 at different temperatures corresponding to the FE-I, FE-IC and FE-II phases, respectively Interatomic distances are given in and bond angles in degrees. (Maxmin) provides the difference between the maximum (max) and minimum (min) separation depending on the phase *t* of the modulation in the FE-IC phase. The mean gives the value averaged over *t*. Standard uncertainties are given in parentheses.

	170 K†	139K		100K‡
	Distance	Distance	Maxmin	Distance
O1H1o1	1.02(4)	1.44(2) (mean)	0.25	0.943(15) (A)
		1.32(2) (min)		1.609(15) (B)
		1.57(2) (max)		1.066(14) (C)
				1.467(14) (D)
O2H1o2	0.73(2)	0.91(2) (mean)	0.06	0.863(15) (A)
		0.88(2) (min)		0.796(15) (B)
		0.94(2) (max)		0.840(14) (C)
				0.815(13) (D)
H1o1N1^i^	1.66(4)	1.320(14) (mean)	0.42	1.879(15) (A)
		1.11(2) (min)		1.027(15) (B)
		1.53(2) (max)		1.700(14) (C)
				1.205(14) (D)
H1o2N2^ii^	2.15(2)	1.945(2) (mean)	0.05	1.908(14) (A)
		1.92(2) (min)		2.121(14) (B)
		1.97(2) (max)		1.944(14) (C)
				2.084(14) (D)
O1N1^i^	2.6446(16)	2.629(2) (mean)	0.07	2.6976(14) (A)
		2.586(2) (min)		2.5736(14) (B)
		2.672(2) (max)		2.5974(42) (D)
				2.6726(14) (C)
O2N2^ii^	2.7722(16)	2.763(2) (mean)	0.09	2.7086(15) (A)
		2.715(3) (min)		2.8251(15) (B)
		2.811(3) (max)		2.7264(15) (C)
				2.8062(15) (D)
O1H1o1N1^i^	159(3)	144.8(15) (mean)	9.9	143.8(11) (A)
		139.5(14) (min)		154.4(11) (B)
		149.7(16) (max)		149.3(10) (C)
				152.6(10) (D)
O2H1o2N2^ii^	145(2)	149.2(18) (mean)	5.4	153.7(11) (A)
		146.4(17) (min)		147.5(12) (B)
		152.0(18) (max)		154.5(11) (C)
				147.60(12) (D)

Symmetry codes for N1 and N2 in the structure model at *T* = 139K are: (i) 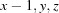; (ii) 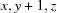.

† From Gotoh *et al.* (2007 ▶).

‡ From Noohinejad *et al.* (2014 ▶); the labels A, B, C and D refer to the four molecular chains, which have become independent in the crystal structure at low temperatures.

**Table 4:** Selected bond lengths () at different temperatures corresponding to the FE-I, FE-IC and FE-II phases, respectively (Maxmin) provides the difference between maximum (max) and minimum (min) separation depending on phase *t* of the modulation in the FE-IC phase. The mean gives the value averaged over *t*. Standard uncertainties are given in parentheses.

	170K†	139K		100K‡
	Distance	Distance	Maxmin	Distance
C3O1	1.2923(13)	1.281(2) (mean)	0.060	1.3200(14) (A)
		1.251(2) (min)		1.2536(14) (B)
		1.311(2) (max)		1.3054(14) (C)
				1.2676(14) (D)
C6O2	1.3204(13)	1.312(3) (mean)	0.004	1.3133(15) (A)
		1.310(2) (min)		1.3204(15) (B)
		1.314(2) (max)		1.3118(14) (C)
				1.3214(14) (D)
C4O3	1.2269(15)	1.219(2) (mean)	0.008	1.2243(14) (A)
		1.215(2) (min)		1.2211(14) (B)
		1.223(2) (max)		1.2248(14) (C)
				1.2201(14) (D)
C1O4	1.2291(15)	1.221(2) (mean)	0.026	1.2183(14) (A)
		1.208(2) (min)		1.2385(14) (B)
		1.234(2) (max)		1.2210(14) (C)
				1.2355(14) (D)
C1C2	1.4404(15)	1.428(3) (mean)	0.043	1.4590(8) (A)
		1.406(3) (min)		1.4114(7) (B)
		1.449(3) (max)		1.4496(8) (C)
				1.4207(8) (D)
C2C3	1.3713(16)	1.372(3) (mean)	0.047	1.3517(9) (A)
		1.349(3) (min)		1.3949(9) (B)
		1.396(3) (max)		1.3622(9) (C)
				1.3843(9) (D)
N1C12	1.3493(13)	1.342(2) (mean)	0.006	1.3451(11) (A)
		1.339(2) (min)		1.3465(11) (B)
		1.345(2) (max)		1.3468(14) (C)
				1.3443(12) (D)
N1C17	1.3472(17)	1.344(2) (mean)	0.005	1.3490(14) (A)
		1.342(2) (min)		1.3505(14) (B)
		1.347(2) (max)		1.3464(14) (C)
				1.3526(14) (D)

† From Gotoh *et al.* (2007 ▶).

‡ From Noohinejad *et al.* (2014 ▶).
