# Supplementary material for: Resonance-stabilized partial proton transfer in hydrogen bonds of incommensurate phenazine–chloranilic acid
Source: Acta Crystallogr B Struct Sci Cryst Eng Mater. 2015 Mar 31;71(Pt 2):228–34. doi: 10.1107/S2052520615004084 (PMC4383393; doi:10.1107/S2052520615004084)
Supplement: Supplementary file 3 [file b-71-00228-sup3.pdf]

Supporting Information

Resonance-stabilized partial proton transfer in hydrogen bonds of incommensurate phenazine–chloranilic acid

Leila Noohinejad,[a] Swastik Mondal,[a,b] Sk Imran Ali,[a] Somnath Dey,[a] Sander van Smaalen[a]* and Andreas Schönleber[a]

[a]*Laboratory of Crystallography, University of Bayreuth, 95440 Bayreuth, Germany, and* [b]*Max-Planck-Institut für Kohlenforschung, Kaiser-Wilhelm-Platz 1, 45470 Mülheim an der Ruhr, Germany. E-mail: smash@uni-bayreuth.de*

Abstract

Supplementary Table 1: atomic coordinates and displacive modulation amplitudes for model A.

Supplementary Table 2: ADPs and their modulation amplitudes for model A.

Supplementary Table 3: bond distances between non-hydrogen atoms for model A.

Supplementary Figures 1–3: different sections of difference Fourier maps.

Table 1. *Fractional atomic coordinates (x, y, z) and amplitudes of the displacement modulation functions (in Å) of model A. Standard uncertainties are given in parentheses.*

Atom	x	y	z	$A_x \times a$	$A_y \times b$	$A_z \times c$	$B_x \times a$	$B_y \times b$	$B_z \times c$
Cl2	0.39426(2)	0.7594	0.425664(9)	0.0027(4)	0.0000	0.0064(3)	-0.0019(4)	-0.0108(3)	0.0012(3)
Cl1	0.10334(2)	1.36699(6)	0.076472(9)	-0.0235(7)	-0.0042(3)	0.0044(5)	-0.0197(9)	-0.0002(3)	0.0023(3)
O1	0.01710(6)	1.0198(3)	0.20084(4)	0.0032(11)	0.0045(1)	-0.0032(13)	-0.0088(11)	-0.0320(1)	0.0069(10)
O2	0.47869(6)	1.1115(3)	0.30175(3)	-0.0354(11)	0.0158(1)	-0.0067(10)	0.0135(16)	-0.0128(1)	0.0016(10)
O3	0.14288(6)	0.7382(3)	0.34754(4)	0.0042(10)	0.0124(2)	-0.0025(12)	-0.0004(10)	-0.0399(1)	0.0043(10)
O4	0.35598(6)	1.3761(3)	0.15557(3)	-0.0174(10)	0.0087(1)	0.0170(10)	0.0069(12)	-0.0113(1)	-0.0010(12)
N1	0.85572(7)	0.6962(3)	0.24458(4)	0.0111(12)	0.0034(1)	0.0293(12)	0.0030(12)	0.0030(1)	-0.0012(15)
N2	0.64611(7)	0.4400(3)	0.25095(4)	-0.0027(14)	0.0015(1)	0.0093(13)	-0.0214(12)	0.0000(1)	0.0043(12)
C1	0.30168(9)	1.2333(3)	0.19627(4)	-0.0437(15)	0.0041(1)	0.0042(13)	-0.0036(21)	-0.0034(1)	0.0003(15)
C2	0.18097(9)	1.2022(3)	0.17186(4)	-0.0191(15)	0.0011(1)	0.0032(13)	-0.0095(16)	-0.0019(1)	0.0022(13)
C3	0.12546(9)	1.0428(3)	0.22157(4)	-0.0104(20)	-0.0019(1)	0.0040(15)	-0.0414(16)	-0.0132(1)	0.0027(13)
C4	0.19362(9)	0.8866(3)	0.30527(4)	0.0022(15)	-0.0030(2)	0.0079(15)	-0.0126(17)	-0.0173 (1)	0.0040(15)
C5	0.31591(8)	0.9219(3)	0.33025(4)	-0.0140(16)	-0.0038(1)	0.0091(13)	-0.0171(15)	-0.0098(1)	0.0020(13)
C6	0.36782(8)	1.0814(3)	0.27986(4)	-0.0334(15)	0.0041(1)	0.0027(13)	-0.0054(19)	-0.0053(1)	0.0007(13)
C7	0.81178(9)	0.5094(3)	0.10230(4)	0.0032(15)	-0.0147(1)	0.0252(17)	-0.0071(15)	0.0026(1)	0.0070(17)
C8	0.73694(9)	0.3530(3)	0.03492(4)	0.0084(15)	-0.0166(1)	-0.0039(17)	0.0028(15)	-0.0060(1)	0.0059(13)
C9	0.63221(9)	0.2204(3)	0.03867(4)	0.0079(15)	-0.0056(1)	-0.0185(15)	0.0069(15)	-0.0075(1)	0.0031(15)
C10	0.60119(9)	0.2457(3)	0.10984(4)	-0.0009(15)	-0.0045(1)	-0.0187(13)	-0.0153(15)	-0.0008(1)	0.0020(15)
C11	0.67723(9)	0.4113(3)	0.18154(4)	-0.0062(17)	-0.0026 (1)	0.0035(17)	-0.0142(15)	0.0015(1)	0.0052(13)
C12	0.78295(8)	0.5441(3)	0.17721(4)	-0.0090(15)	-0.0011(1)	0.0269(17)	-0.0046(15)	0.0038(1)	0.0062(17)
C13	0.69135(9)	0.6285(3)	0.39302(4)	-0.0146(15)	0.0038(1)	0.0214(13)	0.0014(15)	0.0011(1)	-0.0006(15)
C14	0.76521(9)	0.7854(3)	0.46057(4)	-0.0145(15)	-0.0072(1)	0.0135 (15)	0.0148(15)	0.0056(1)	-0.0049(15)
C15	0.87021(9)	0.9210(3)	0.45697(5)	0.0000 (15)	-0.0038(1)	0.0008(17)	0.0090(16)	0.0132(1)	-0.0059(15)
C16	0.90122(9)	0.8951(3)	0.38591(5)	-0.0010(16)	-0.0049(1)	0.0056(15)	0.0235(17)	0.0151(1)	-0.0051(15)
C17	0.82595(9)	0.7284(3)	0.31464(4)	-0.0131(16)	-0.0030(1)	0.0125(15)	0.0121(15)	0.0034(1)	-0.0008(15)
C18	0.71946(9)	0.5983(3)	0.31757(4)	-0.0209(15)	0.0030(1)	0.0155(13)	-0.0047(16)	0.0019(1)	0.0007(15)
H1o1	-0.0461(11)	0.765(4)	0.2321(8)	0.1868(15)	0.0527(1)	0.0168(15)	-0.0792(15)	-0.0113(2)	0.0075(13)
H1o2	0.5251(11)	1.158(4)	0.2704(8)	-0.0074(15)	0.0151(1)	-0.0101(15)	0.0000(15)	-0.0113(1)	-0.0064(15)
H7	0.8834	0.5955	0.0992	0.0062	-0.0211	0.0471	-0.0074	0.0098	0.0038
H8	0.7558	0.3327	-0.0161	0.0087	-0.0248	-0.0067	0.0099	-0.0079	0.0064
H9	0.5814	0.1096	-0.0098	-0.0012	0.0011	-0.0219	0.0161	-0.0136	-0.0004
H10	0.5296	0.1537	0.1115	0.0074	-0.0030	-0.0168	-0.0210	0.0064	-0.0023
H13	0.6202	0.5382	0.3962	-0.0148	0.0053	0.0185	-0.0074	0.0053	0.0011
H14	0.7459	0.8045	0.5115	-0.0161	-0.0072	0.0067	0.0173	0.0090	-0.0053
H15	0.9207	1.0334	0.5054	0.0012	0.0072	-0.0135	0.0025	0.0196	-0.0053
H16	0.9727	0.9881	0.3842	0.0111	0.0041	-0.0050	0.0186	0.0188	-0.0056

Table 2. *Anisotropic atomic displacement parameters (anisotropic ADPs) U_{ij} of model A. For each atom are given the basic-structure value (first line), the sine amplitude of the first-harmonic modulation function (sin; second line), and the cosine amplitude (cos; third line). Standard uncertainties are given in parentheses.*

Atom		U_{11}	U_{22}	U_{33}	U_{12}	U_{13}	U_{23}
Cl2		0.01737(18)	0.01914(12)	0.01152(7)	-0.00017(11)	0.00280(9)	0.00293(6)
	sin	0.0032(2)	-0.00022(16)	0.00007(18)	0.00000(15)	0.00024(17)	0.00049(11)
	cos	0.0021(2)	-0.00196(17)	-0.00069(18)	0.00006(14)	-0.00065(16)	0.00021(11)
Cl1		0.01772(18)	0.01883(12)	0.01129(7)	0.00005(11)	0.00285(9)	0.00285(6)
	sin	0.0017(2)	-0.00089(16)	-0.00044(17)	-0.00150(17)	-0.00058(16)	0.00029(12)
	cos	-0.0007(2)	-0.00023(16)	-0.00047(18)	-0.00050(17)	-0.00097(17)	0.00067(11)
O1		0.0117(4)	0.0259(4)	0.0183(2)	-0.0023(4)	0.0042(3)	0.0039(3)
	sin	0.0026(7)	0.0004(5)	-0.0007(6)	0.0002(4)	0.0012(5)	0.0001(4)
	cos	0.0037(7)	-0.0035(6)	0.0001(6)	-0.0010(4)	0.0010(5)	0.0020(4)
O2		0.0132(4)	0.0253(4)	0.0169(2)	-0.0030(4)	0.0056(3)	0.0055(3)
	sin	0.0001(7)	0.0037(5)	0.0002(6)	-0.0028(5)	-0.0007(5)	-0.0008(4)
	cos	0.0009(7)	-0.0024(5)	-0.0004(6)	0.0002(4)	-0.0002(5)	0.0008(4)
O3		0.0167(4)	0.0272(4)	0.0177(2)	-0.0030(5)	0.0081(3)	0.0054(3)
	sin	-0.0025(7)	0.0019(6)	0.0002(6)	0.0008(4)	0.0008(5)	0.0003(4)
	cos	0.0055(7)	-0.0061(6)	-0.0011(6)	0.0007(5)	0.0001(5)	0.0029(4)
O4		0.0160(4)	0.0254(4)	0.0165(2)	-0.0021(5)	0.0067(3)	0.0054(3)
	sin	0.0002(7)	0.0019(5)	-0.0009(6)	-0.0007(5)	0.0003(5)	0.0012(4)
	cos	0.0013(7)	-0.0011(6)	-0.0002(6)	0.0012(4)	0.0018(5)	-0.0007(4)
N1		0.0132(5)	0.0140(3)	0.0123(2)	0.0001(4)	0.0029(3)	0.0007(2)
	sin	-0.0004(8)	0.0000(5)	-0.0016(6)	-0.0002(5)	-0.0029(6)	0.0022(4)
	cos	-0.0024(8)	0.0013(5)	-0.0001(6)	-0.0012(5)	-0.0016(6)	-0.0014(4)
N2		0.0133(5)	0.0142(3)	0.0127(2)	-0.0009(4)	0.0037(3)	0.0005(2)
	sin	0.0020(8)	-0.0005(5)	0.0005(6)	-0.0003(5)	0.0002(6)	0.0005(4)
	cos	-0.0007(7)	0.0005(5)	-0.0001(6)	-0.0015(5)	-0.0019(5)	0.0016(4)
C1		0.0165(6)	0.0137(4)	0.0114(2)	-0.0004(5)	0.0044(3)	0.0002(3)
	sin	0.0107(10)	0.0011(6)	-0.0032(7)	-0.0030(6)	-0.0016(7)	0.0006(4)
	cos	-0.0030(10)	-0.0001(6)	0.0004(7)	0.0006(6)	-0.0010(7)	-0.0003(4)
C2		0.0144(6)	0.0152(4)	0.0107(2)	-0.0016(4)	0.0035(3)	0.0005(2)
	sin	-0.0024(10)	0.0019(6)	-0.0020(7)	-0.0020(6)	-0.0024(7)	-0.0002(4)
	cos	-0.0025(9)	-0.0001(6)	-0.0004(7)	0.0004(6)	-0.0013(7)	0.0009(4)
C3		0.0145(6)	0.0152(4)	0.0122(2)	-0.0001(5)	0.0039(3)	0.0000(3)
	sin	0.0014(10)	0.0000(6)	-0.0009(8)	-0.0020(6)	-0.0001(7)	0.0005(5)
	cos	0.0026(10)	-0.0031(6)	-0.0002(7)	-0.0029(6)	0.0004(7)	0.0010(4)
C4		0.0143(6)	0.0150(4)	0.0120(2)	-0.0011(5)	0.0050(3)	-0.0004(3)
	sin	0.0006(9)	0.0001(6)	0.0012(7)	0.0022(6)	0.0020(7)	-0.0003(5)
	cos	0.0042(10)	-0.0029(7)	-0.0015(8)	-0.0015(6)	-0.0016(7)	0.0003(4)
C5		0.0125(6)	0.0137(4)	0.0110(2)	-0.0009(4)	0.0029(3)	0.0007(2)
	sin	-0.0053(9)	-0.0006(6)	0.0021(7)	0.0016(6)	0.0004(7)	0.0030(4)
	cos	-0.0004(9)	-0.0005(6)	-0.0015(7)	-0.0010(6)	-0.0005(6)	-0.0003(4)
C6		0.0110(6)	0.0137(4)	0.0118(2)	-0.0009(4)	0.0028(3)	0.0002(2)
	sin	0.0030(9)	0.0002(6)	0.0003(7)	-0.0023(6)	-0.0003(7)	0.0008(4)
	cos	0.0059(10)	-0.0002(6)	-0.0011(7)	0.0007(5)	-0.0005(7)	0.0008(4)

Continued on next page.

Continued from previous page.

Atom		U_{11}	U_{22}	U_{33}	U_{12}	U_{13}	U_{23}
C7		0.0159(6)	0.0159(4)	0.0142(3)	-0.0004(5)	0.0051(3)	-0.0009(3)
	sin	0.0032(10)	-0.0017(6)	-0.0005(7)	0.0001(6)	-0.0012(7)	0.0022(5)
	cos	0.0021(9)	-0.0011(6)	0.0007(7)	0.0016(6)	-0.0005(7)	0.0020(5)
C8		0.0187(6)	0.0179(4)	0.0139(2)	0.0045(5)	0.0055(3)	0.0000(3)
	sin	0.0037(11)	-0.0040(7)	0.0009(8)	-0.0013(6)	0.0009(7)	-0.0001(5)
	cos	0.0002(10)	-0.0024(6)	0.0020(7)	-0.0012(6)	0.0009(7)	0.0030(5)
C9		0.0166(6)	0.0165(4)	0.0138(3)	0.0015(5)	0.0022(3)	-0.0020(3)
	sin	0.0048(10)	0.0002(6)	-0.0014(8)	-0.0003(6)	0.0009(7)	-0.0007(5)
	cos	0.0142(10)	-0.0009(6)	-0.0038(8)	0.0013(6)	0.0024(7)	0.0006(5)
C10		0.0148(6)	0.0164(4)	0.0137(2)	-0.0047(5)	0.0023(3)	-0.0020(3)
	sin	-0.0042(9)	0.0009(6)	-0.0004(7)	-0.0022(6)	-0.0014(7)	-0.0020(5)
	cos	-0.0011(10)	0.0003(6)	-0.0004(8)	0.0012(6)	-0.0034(7)	0.0024(5)
C11		0.0161(6)	0.0119(4)	0.0118(2)	0.0000(4)	0.0028(3)	0.0008(2)
	sin	0.0037(11)	-0.0007(6)	0.0003(8)	-0.0011(5)	-0.0007(7)	-0.0005(4)
	cos	-0.0014(9)	0.0000(6)	-0.0008(7)	-0.0008(6)	0.0000(6)	0.0021(4)
C12		0.0112(5)	0.0127(4)	0.0130(2)	0.0023(4)	0.0038(3)	0.0018(2)
	sin	0.0023(9)	-0.0002(6)	-0.0006(7)	-0.0002(5)	0.0001(6)	0.0018(4)
	cos	0.0015(9)	0.0004(6)	0.0007(7)	-0.0022(5)	-0.0005(7)	0.0014(4)
C13		0.0159(6)	0.0180(4)	0.0135(2)	0.0018(5)	0.0060(3)	0.0012(3)
	sin	0.0045(10)	0.0005(6)	-0.0020(7)	0.0004(6)	-0.0011(7)	0.0016(5)
	cos	-0.0003(9)	0.0007(7)	0.0000(7)	-0.0013(6)	-0.0003(7)	0.0002(5)
C14		0.0200(6)	0.0177(4)	0.0143(3)	0.0006(5)	0.0057(3)	-0.0027(3)
	sin	-0.0021(10)	-0.0015(7)	-0.0002(8)	-0.0022(6)	-0.0019(7)	0.0012(5)
	cos	-0.0012(10)	-0.0008(6)	0.0024(8)	-0.0023(6)	0.0019(7)	-0.0014(5)
C15		0.0160(6)	0.0168(4)	0.0143(3)	0.0002(5)	0.0024(3)	-0.0028(3)
	sin	-0.0004(9)	0.0002(6)	-0.0020(7)	0.0017(6)	-0.0018(7)	-0.0007(5)
	cos	0.0044(11)	0.0025(6)	-0.0004(8)	0.0021(6)	0.0010(7)	-0.0010(5)
C16		0.0126(6)	0.0144(4)	0.0144(2)	0.0010(5)	0.0009(3)	-0.0006(3)
	sin	0.0023(8)	-0.0013(6)	0.0000(7)	-0.0005(6)	-0.0007(6)	0.0001(5)
	cos	-0.0013(11)	0.0014(6)	0.0017(8)	0.0011(5)	0.0009(7)	-0.0012(4)
C17		0.0144(6)	0.0124(4)	0.0124(2)	0.0018(4)	0.0050(3)	0.0009(2)
	sin	0.0049(9)	-0.0011(6)	-0.0005(7)	-0.0002(6)	0.0007(7)	0.0012(4)
	cos	-0.0060(9)	0.0003(6)	0.0011(7)	0.0032(5)	-0.0014(7)	0.0001(4)
C18		0.0151(6)	0.0117(4)	0.0117(2)	-0.0009(4)	0.0023(3)	0.0007(2)
	sin	0.0015(9)	-0.0001(6)	-0.0012(7)	-0.0013(5)	-0.0005(7)	0.0021(4)
	cos	-0.0001(9)	0.0010(6)	-0.0009(7)	-0.0012(6)	0.0000(6)	0.0005(4)

Table 3. *Bond lengths (Å) for bonds between non-hydrogen atoms in model A. Standard uncertainties are given in parentheses.*

Bond	Mean	Min.	Max.	Max-Min
Cl2-C5	1.7153(15)	1.7131(15)	1.7178(15)	0.0047
Cl1-C2	1.7155(15)	1.7119(15)	1.7190(15)	0.0071
O1-C3	1.281(2)	1.251(2)	1.311(2)	0.060
O2-C6	1.312(3)	1.310(2)	1.314(2)	0.004
O3-C4	1.219(2)	1.215(2)	1.223(2)	0.008
O4-C1	1.221(2)	1.208(2)	1.234(2)	0.026
C1-C2	1.428(3)	1.406(3)	1.449(3)	0.043
C1-C6	1.508(2)	1.505(2)	1.510(2)	0.005
C2-C3	1.372(3)	1.349(3)	1.396(3)	0.047
C3-C4	1.522(2)	1.507(2)	1.537(2)	0.030
C4-C5	1.447(3)	1.438(3)	1.456(3)	0.018
C5-C6	1.351(3)	1.346(3)	1.356(3)	0.010
N1-C12	1.342(2)	1.339(2)	1.345(2)	0.006
N1-C17	1.344(2)	1.342(2)	1.347(2)	0.005
N2-C11	1.342(2)	1.338(2)	1.345(2)	0.007
N2-C18	1.3470(19)	1.342(2)	1.352(2)	0.010
C7-C8	1.360(2)	1.355(2)	1.364(2)	0.009
C7-C12	1.417(3)	1.415(3)	1.420(3)	0.005
C8-C9	1.408(3)	1.403(3)	1.413(3)	0.010
C9-C10	1.368(3)	1.364(3)	1.373(3)	0.009
C10-C11	1.427(2)	1.420(2)	1.434(2)	0.014
C11-C12	1.423(3)	1.420(3)	1.426(3)	0.006
C13-C14	1.357(2)	1.351(2)	1.363(2)	0.012
C13-C18	1.420(3)	1.417(3)	1.423(3)	0.006
C14-C15	1.414(3)	1.405(3)	1.424(3)	0.019
C15-C16	1.367(3)	1.361(3)	1.372(3)	0.011
C16-C17	1.420(2)	1.417(2)	1.424(2)	0.007
C17-C18	1.421(3)	1.417(3)	1.424(3)	0.007

1. Modulation of the acidic hydrogen atoms

The difference Fourier map obtained after the final refinement of model A reveals maxima on the covalent bonds (Fig. 1). Apparently, the resolution of the diffraction data (Table 1 in the main text) was sufficient to obtain the reorganisation of electron density due to chemical bonding. Nevertheless, the resolution of the diffraction data is worse and the temperature of the crystal is higher than is generally accepted as necessary for a multipole refinement. Furthermore, software is not available for multipole refinements of modulated structures. Therefore, we present model A in the independent-atom approximation as the best model that we have been able to achieve.

Similarly to covalent bonds, the difference Fourier map reveals density at lone pairs of oxygen atoms. This density interferes with the density of the modulated hydrogen atoms. Nevertheless, the difference Fourier map obtained after refinement of a model without the acidic hydrogen atoms clearly shows positive density near O2 of the O2–H1o2$\cdots$N2 hydrogen bond, which is well described by introducing a virtually non-modulated hydrogen atom. See top row *vs* bottom row in Fig. 2. The remaining density near O2 in the difference Fourier map of model A will represent the lone pair of O2.

Sections through the O1–H1o1$\cdots$N1 hydrogen bond of the same two difference Fourier maps reveal density close to N1 for $t = 0.31$ and density close to O1 for $t = 0.85$ (bottom row in Fig. 3). This density is well described by the modulated positions of H1o1 hydrogen atom, while the remaining density near O1 in the difference Fourier map of model A should be interpreted as the lone pair of O1.

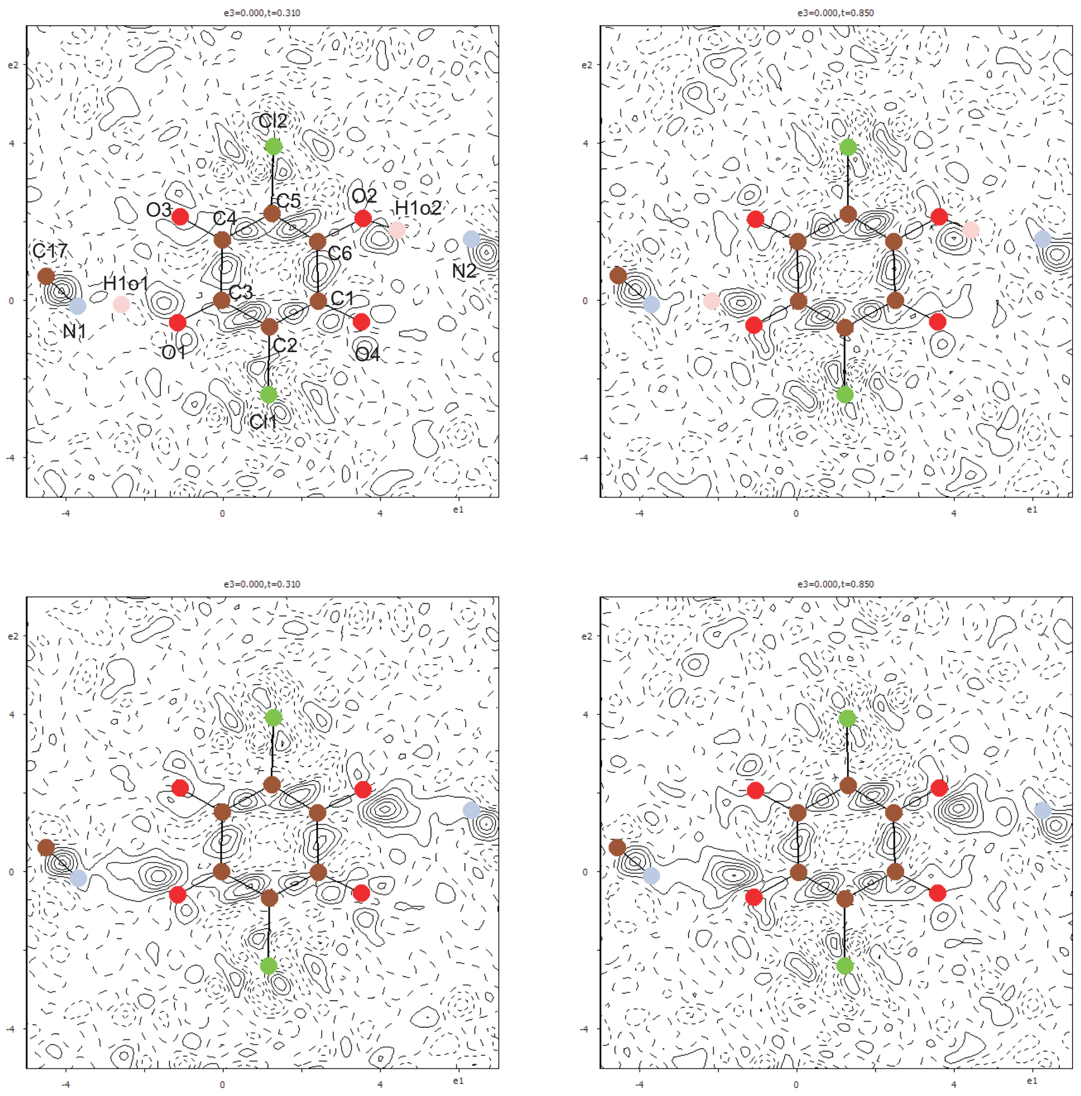

Fig. 1. Difference Fourier maps showing a section of 12×12 Å^2 in the plane through the atoms C1—C3—C5 of the chloranilic acid ring.
Top row: model A.
Bottom row: model A without the acidic hydrogen atoms.
Left: section $t = 0.31$ corresponding to a maximum of the distance O1–H1o1 (*cf* Fig. 3 in the main text).
Right: section $t = 0.85$ corresponding to a minimum of the distance O1–H1o1.
Positive contours (solid lines), negative contours (dotted lines) and the zero contour (dashed lines) are drawn at intervals of 0.1 electrons/Å^3. Coloured circles are the projected positions of atoms that appear within 0.42 Å from the plane.

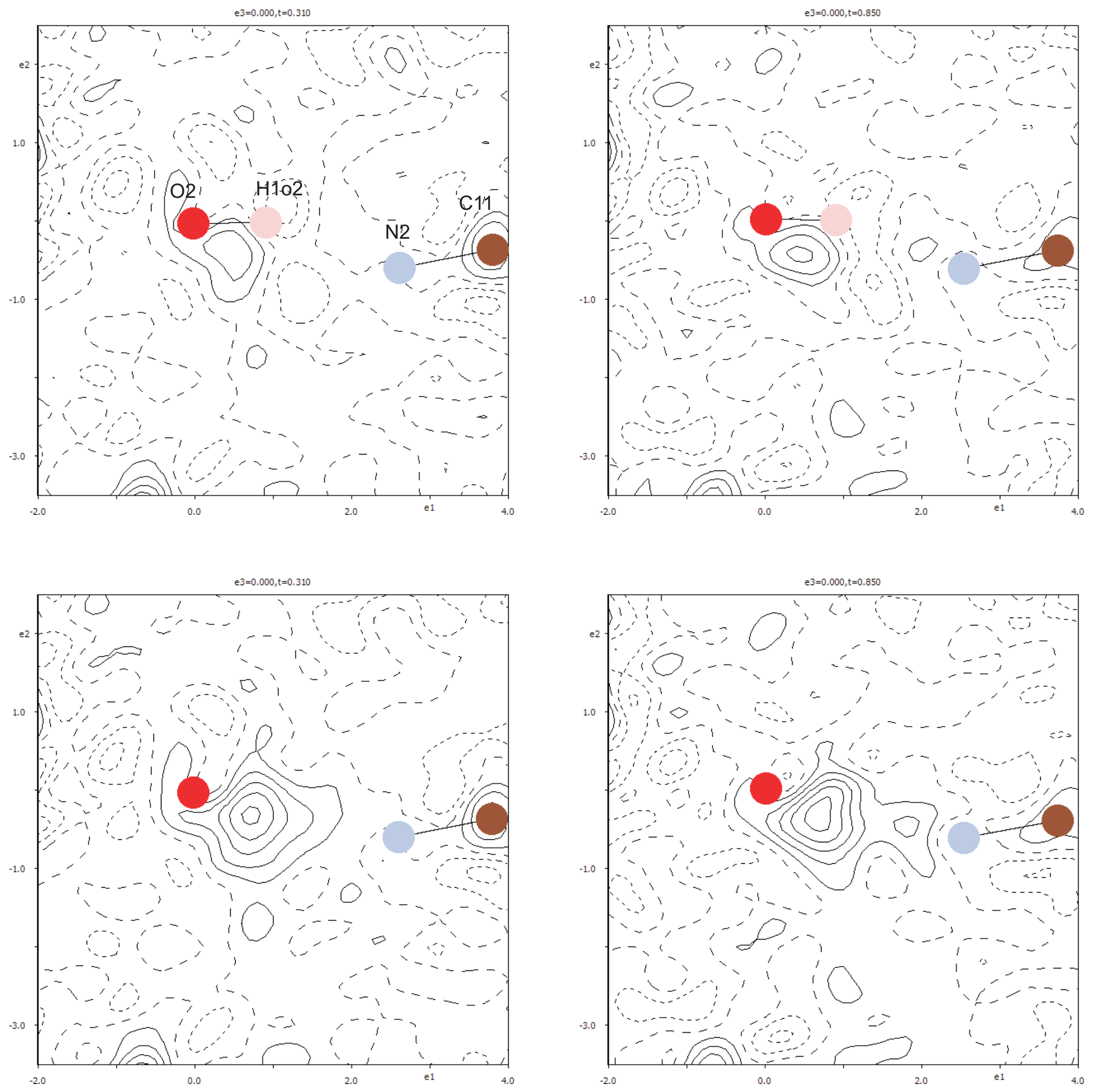

Fig. 2. Difference Fourier maps showing a section of 6×6 Å^2 in the plane through the hydrogen bond defined by the atoms O2—H1o2···N2.
Top row: model A.
Bottom row: model A without the acidic hydrogen atoms.
Left: section $t = 0.31$ corresponding to a maximum of the distance O1–H1o1 (*cf* Fig. 3 in the main text).
Right: section $t = 0.85$ corresponding to a minimum of the distance O1–H1o1.
Positive contours (solid lines), negative contours (dotted lines) and the zero contour (dashed lines) are drawn at intervals of 0.1 electrons/Å^3. Coloured circles are the projected positions of atoms that appear within 0.42 Å from the plane.

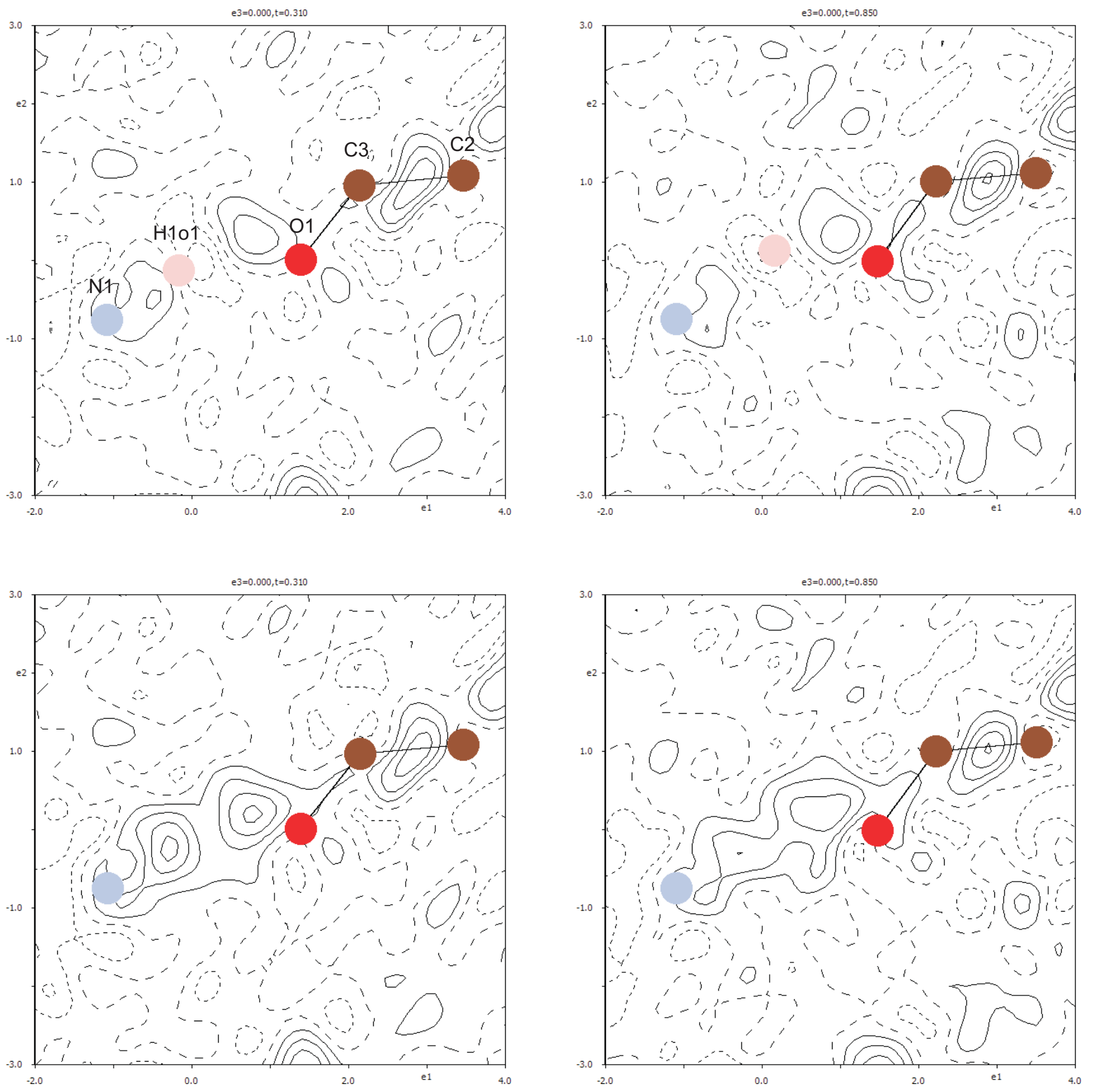

Fig. 3. Difference Fourier maps showing a section of 6×6 Å^2 in the plane through the hydrogen bond defined by the atoms O1—H1o1···N1.
Top row: model A.
Bottom row: model A without the acidic hydrogen atoms.
Left: section $t = 0.31$ corresponding to a maximum of the distance O1–H1o1 (*cf* Fig. 3 in the main text).
Right: section $t = 0.85$ corresponding to a minimum of the distance O1–H1o1.
Positive contours (solid lines), negative contours (dotted lines) and the zero contour (dashed lines) are drawn at intervals of 0.1 electrons/Å^3. Coloured circles are the projected positions of atoms that appear within 0.42 Å from the plane.
